# Influence of biochemical diagnosis of growth hormone deficiency on replacement therapy response and retesting results at adult height

**DOI:** 10.1038/s41598-021-93963-6

**Published:** 2021-07-15

**Authors:** Giulia Rodari, E. Profka, F. Giacchetti, I. Cavenaghi, M. Arosio, C. Giavoli

**Affiliations:** 1 Endocrinology Unit, Fondazione IRCCS Ca’ Granda Ospedale Maggiore Policlinico, Via Francesco Sforza 28, 20122 Milan, Italy; 2 Department of Clinical Sciences and Community Health, University of Milan, Milan, Italy

**Keywords:** Growth disorders, Pituitary diseases

## Abstract

Isolated growth hormone deficiency (IGHD) is the most frequent endocrinological disorder in children with short stature, however the diagnosis is still controversial due to the scarcity of reliable diagnostic criteria and pre-treatment predictive factors of long term-response. To evaluate recombinant growth hormone (rGH) long-term response and retesting results in three different groups of children divided in accordance with the biochemical criteria of initial diagnosis. Height gain (∆HT) at adult height (AH) and retesting results were evaluated in 57 rGH treated children (M = 34, 59.6%) divided into 3 groups according to initial diagnosis: Group A (n = 25) with max GH peak at stimulation test < 8 µg/L, Group B (n = 19) between 8 and 10 µg/L and Group C (n = 13) with mean overnight GH < 3 µg/L (neurosecretory dysfunction, NSD). Retesting was carried out in all patients after at least one month off therapy upon reaching the AH. 40/57 (70.2%) patients were pre-pubertal at diagnosis and showed ∆HT of 1.37 ± 1.00 SDS, with no significant differences between groups (*P* = 0.08). Nonetheless, 46% patients in Group B showed ∆HT < 1SDS (vs 13% and 12% in Group A and C, respectively) and 25% children failed to reach mid-parental height (vs 6% and 0% in Group A and C, respectively). At AH attainment, IGHD was reconfirmed in 28% (7/25) and 10% (2/19) in Group A and B, respectively. A reduction of diagnostic cut-off at GH stimulation tests could better discriminate between “good” and “poor responders” and predict the persistence of IGHD through transition. Group C response and the predictive value of baseline IGF-I SDS bring back to light NSD: should we consider an underlying hypothalamic derangement when the clinical presentation is strongly consistent with IGHD but pharmacological stimulation test is normal?

## Introduction

Growth hormone deficiency (GHD) is the most common endocrine disorder in children with short stature, with a reported prevalence of 1 in 4000^1^. Although relatively rare, a correct diagnosis is crucial, as recombinant growth hormone (rGH) replacement therapy is highly effective in GHD, helping patients to achieve a normal adult height (AH) according to their genetic potential^2^. On the other hand, a false positive diagnosis can lead to a significant wasted expenditure and unnecessary exposure to rare potential adverse effects.

According to the Consensus Guidelines^2, 3^, the clinician should synthesize auxological, biochemical and neuroradiological data to attain GHD diagnosis. However, until now there are significant controversies, especially from the laboratory point of view. In fact, though growth hormone (GH) provocative tests play a key role in the diagnostic process, measured GH concentration can vary significantly according to stimulation test or GH assay used. In order to distinguish between GHD and non-GHD patients, a cut-off of GH concentration should be established but to date, in the lack of any “gold standard test” for GHD diagnosis in childhood, any cut-off level seems somehow arbitrary. Moreover, GH secretion should be considered as a continuum from normality to severe GHD, implying a physiological overlap in peak GH concentrations between children with idiopathic short stature (ISS) and those with GHD. Due to these issues, separating the group of ISS from mild GHD can be challenging: the reproducibility of provocative tests indeed is extremely poor and a great number of falsely abnormal responses are frequently observed even in normal children^4^.

Even considering auxological criteria first, diagnosis could be challenging. When GH provocative tests were first introduced in the 1960s, the biochemical diagnosis of GHD in childhood was defined for GH peak < 5 µg/L^5^. Over time, this cut-off has been increased on the basis of very limited evidence to 10 µg/L^6^. Nonetheless, the latest Guidelines recognize that this threshold should be revised according to the advent of monoclonal antibody testing and newer standards that produce GH measurements approximately 40% lower than those obtained with the oldest immunoassays^2, 3, 7, 8^. Therefore, the cut-offs for GHD should be reduced in the attempt to minimize the large number of false-positive results that lead to misclassify as deficient a child with normal GH secretion^9–16^.

Adjustment of this threshold should be defined locally by the paediatric endocrinology society specific to the country or region. In Italy, rGH prescription and reimbursement is regulated by Note 39 of the Italian Drugs Agency (AIFA). Its revision, in 2014, reducing the cut-off at provocative tests from 10 to 8 µg/L, can be considered an effort in that sense^17^.

Nonetheless, there are rare patients who appear to have true GHD even though their stimulated GH peak exceeds traditional cut-offs or patients diagnosed having GHD in the presence of a peak GH between 8 to 10 µg/L showing a good response to rGH therapy.

As above mentioned, the reproducibility of stimulation tests is extremely poor^18, 19^, consequently, in the past, the assessment of physiological GH secretion using the 12-h or the 24-h overnight GH profile with blood sampling every 20 min was considered a possible alternative for biochemical GHD diagnosis. Differently from provocative tests, GH profile has a superior reproducibility^9, 20–22^ but a lower sensitivity, failing to diagnose 57% of GHD patients identified by GH stimulation tests^20^. The presence of an abnormal GH profile (reduced number of GH pulses and reduced pulse amplitude) along with low IGF-I concentration, auxology compatible with a diagnosis of GHD, bone age delay of at least 2 years and a normal GH stimulation test, define growth hormone neurosecretory dysfunction (NSD), condition probably due to hypothalamic derangement.

However, rGH therapy is no longer indicated in NSD, according to the strong recommendation against the use of spontaneous GH secretion in the diagnosis of GHD published in the Consensus Guidelines of 2016^2^.

Moreover, at the end of growth, up to 60–80% of patients diagnosed with isolated GHD (IGHD) re-test normal^23–27^. It is unknown yet if this represents a form of transient GHD or a false positive diagnosis during childhood.

Given these open issues about biochemical diagnosis of IGHD in childhood, the aim of the present retrospective, single-centre study was to assess rGH long-term response (gain in height at AH) and retesting results in three different groups of children divided in accordance with the biochemical criteria of initial diagnosis.

## Materials and methods

### Patients and study protocol

The study included 57 children (M = 34, 59.6%) treated with rGH at the Endocrinology Unit of Fondazione IRCCS Ca’ Granda Ospedale Maggiore Policlinico of Milan until AH, from 1993 to 2019 for IGHD or NSD. At the time of diagnosis, all patients fulfilled the auxological criteria of height (HT) ≤ − 3 SDS OR HT ≤ − 2 SDS with a growth velocity (GV) ≤ − 1 SDS OR a GV ≤ − 2 SDS for the last year or GV ≤ − 1.5 SDS for two consecutive years. At baseline age, mid-parental height (MPH), HT, weight, body mass index (BMI) and pubertal stage were recorded and SDS values for HT and MPH were calculated according to the Italian reference charts for Italian patients and the WHO growth charts for others (n = 6) according to “Growth calculator 4” by Italian Society of Pediatric Endocrinology and Diabetology (ISPED-SIEDP) available online^28^. Body mass index SDS was evaluated according to the WHO specific charts^28^. Before rGH treatment, bone age was calculated according to the standards of Tanner-Whitehouse^29^ and a brain MRI was performed in every patient. All patients underwent two of the following GH stimulation tests: clonidine, arginine, glucagon or insulin tolerance test (ITT). In cases of suspected NSD with normal response to GH stimulation test, an overnight GH profile (12 h) was carried out with samples taken with a short intravenous catheter every 30 min from 20.00 to 08.00^9, 30^.

In order to avoid biases due to late diagnosis and treatment, AH response was analysed separately in the 40/57 patients who were pre-pubertal at rGH start.

At AH attainment, each patient was retested with GHRH + arginine (n = 54) or ITT (n = 3) after at least one month off therapy. The lowest GH cut-off limit at retesting considered normal was 19 µg/L for the combined test and 6 µg/L for ITT^31, 32^.

Patients with other pituitary hormone deficiencies, GHD secondary to neoplasia, irradiation or pituitary stalk interruption syndrome were excluded from the study as well as patients born small for gestational age or with underlying chronic diseases (i.e. coeliac disease).

According to biochemical diagnostic criteria of Note AIFA 39, until 2009 rGH therapy could be reimbursed to children with either two abnormal provocative tests with a cut-off of 10 µg/L or NSD, from 2009 rGH treatment was no more reimbursed in NSD, while in 2014 GHD the cut-off was lowered to 8 µg/L. In line with those changes, our patients were divided into three groups: Group A (n = 25) of IGHD patients with max GH peak at both stimulation tests < 8 µg/L, Group B (n = 19) of IGHD patients with a max GH peak between 8 µg/L and 10 µg/L at least at one test and Group C (n = 13) classified as NSD according to overnight GH profile (mean GH < 3 µg/L) but peak GH > 10 µg/L at provocative test^33, 34^.

Poor response at AH was defined in the presence of total gain ∆HT < 1 SDS^35^. Mid-parental height target achievement was defined as AH-MPH > − 2 SDS.

All procedures performed in this study were in accordance with the ethical standards of the institutional research committee and with the 1964 Helsinki declaration and its later amendments or comparable ethical standards. Informed written consent was obtained from all study subjects and, under 18 years of age, from parents and/or a legal guardian.

### Assay methods

From 1992 to October 2007, a two-site monoclonal immunofluorometric assay method (AutoDelfia kit, Wallac, Inc. OY, Turku, Finland) was used. The sensitivity of this method was 0.01 µg/L and intra- and interassay coefficients of variation were 2% and 1.7%, respectively. After October 2007, GH was assayed with a chemiluminescence method (Immulite 2000, Siemens Medical Solutions Diagnostics, Los Angeles, CA) with a detection limit of 0.01 µg/L. In both cases, the standards were calibrated to the first World Health Organization International Reference Preparation (code 80/505). After the second semester of 2010, the standards of the Immulite method were calibrated to the WHO International Standard IS 98/574.

Serum IGF-I concentrations were measured by commercial RIA kits starting from 1985. According to the RIA assay used before 1996 (Incstar, Stillwater, MN), the removal of binding proteins was obtained by acidification and subsequent filtration on ODS C18 cartridges. The intra- and interassay coefficients of variation were 15 and 16%, respectively. Afterwards and until 2008, IGF-I levels were assessed by the commercial radioimmunometric assay kit of Mediagnost (Tübingen, Germany). The separation of IGF-I from binding proteins was obtained by acidification in IGF-II excess, and IGF-II cross-reactivity was less than 0.05%. The intra- and interassay coefficients of variation were 3.2 and 8.9%, respectively. After 2008, IGF-I levels were measured by a chemiluminescent immunometric assay (Immulite 2000 IGF-I; Siemens Medical Solutions Diagnostics, Los Angeles, CA), with an intra- and interassay coefficient of variation of 2.9 and 7.4%, respectively. Standards used for calibration were IRR 87/518 to April 2017 and IS 02/254 from May 2017. The values were compared with those from an appropriate age- and sex-adjusted range for each kit and expressed in standard deviation scores (SDS).

### Statistical analysis

Statistical analysis was performed using SPSS version 26 statistical package (SPSS IBM, New York, USA).

Descriptive analysis was used to characterise the study population (mean and standard deviation for normally distributed continuous variables, median and range for others). To compare two normally distributed continuous variables, Student’s t test was used; otherwise, a Mann–Whitney test was employed. In order to compare the three groups at baseline and at AH, for normally distributed continuous variables was performed one-way ANOVA test, otherwise Kruskal–Wallis test was used. Categorical variables were compared with the χ^2^ or Fisher’s exact test. Pearson’s correlation coefficient was used as a measure of the linear relationship between continuous variables. Multiple regression analysis was used to assess the importance of various auxological, treatment and GH secretion variables in the prediction of growth response (age at start, HT SDS, MPH difference, BMI SDS and IGF-I SDS at baseline, GH peak, rGH dose during the first and the last year of therapy and duration of treatment). Stepwise removal was performed with exclusion criterion *P* > 0.10. Statistical significance was defined as a two-sided *P* < 0.05.

### Ethical approval

Ethical approval was waived by the local Ethics Committee of Fondazione IRCCS Ca' Granda Ospedale Maggiore Policlinico of Milan. In view of the retrospective nature of the study and all the procedures being performed were part of the routine care.

## Results

### Baseline characteristics

Median age at GHD diagnosis was 11.9 years (range 1.9–17.1 years), being not significantly different in the three groups (*P* = 0.12). Among them, 34 (59.6%) patients were males (13/25 in Group A, 13/19 in Group B and 8/13 in Group C, respectively. *P* = 0.48). Bone age was available for 43/57 patients and was 9.0 ± 3.1 years, not significantly different in the three groups (*P* = 0.07)*.* The majority of them was pre-pubertal (40/57, 70.2%) at rGH start.

Baseline characteristics were not different in the three Groups as stated in Table 1.

**Table 1:** Baseline characteristics expressed as mean (SD) or median (min–max).

	Patients (57)	Group A (25)	Group B (19)	Group C (13)	Sign	N
CA (years)	11.9 (1.9–17.1)	12.9 (2.8–15.2)	11.6 (6.4–17.1)	10.5 (1.9–15.7)	*P* = 0.12	56
MPH (SDS)	−0.86 (0.82)	−0.76 (0.90)	−0.82 (0.74)	−1.12 (0.77)	*P* = 0.47	53
HT (SDS)	−2.48 (0.77)	−2.36 (0.86)	−2.43 (0.64)	−2.82 (0.72)	*P* = 0.26	53
BMI (SDS)	0.11 (1.36)	0.14 (1.46)	−0.08 (1.24)	0.44 (1.41)	*P* = 0.62	52
IGF-I (SDS)	−1.68 (0.86)	−1.67 (0.87)	−1.72 (0.63)	−1.61 (1.17)	*P* = 0.94	53

*CA* chronological age, *MPH* mid-parental height, *HT* height, *BMI* body mass index, *IGF-I* insulin-like growth factor-I.

At GH stimulation test (arginine, glucagon, ITT and clonidine), median max GH peak was 5.5 µg/L (range 0.1–7.9 µg/L) in Group A and 8.6 µg/L (range 8.0–9.9 µg/L) in Group B (*P* < 0.0001). In Group C mean GH at overnight profile was 1.7 ± 0.9 µg/L (range 0.5–2.8 µg/L).

Brain MRI was normal in 61% patients (35/57), whereas 15/57 patients (26%, 7 Group A, 5 Group B, 3 Group C, respectively) had an anterior pituitary hypoplasia, 6/57 (11%, 5 Group A, 1 Group B, respectively) a partial empty sella and 1/57 belonging to Group A (2%) a pars intermedia cyst.

Growth hormone therapy was started at a mean dose of 0.028 ± 0.007 mg/kg/day, being higher in Group C (0.027 ± 0.007, 0.025 ± 0.006, 0.035 ± 0.005 mg/kg/day, in Group A, B and C respectively, *P* = 0.002), even if in the replacement range.

### Adult height and retesting results

After a mean time of treatment of 4.7 ± 2.7 years (not significantly different in the three groups, *P* = 0.16), the mean AH was 163.3 ± 9.0 cm, − 1.22 ± 0.89 SDS, not significantly different in the three groups (median AH of − 0.90, − 1.29 and − 1.65 SDS in Group A, B and C, respectively, *P* = 0.14) with an overall height gain (ΔHT) at AH of 1.22 ± 1.04 SDS (*P* < 0.0001 *vs* baseline; median ΔHT of 1.17, 1.0, 1.31 SDS in Group A, B and C, respectively, *P* = 0.27). At AH, the MPH difference (MPH SDS-AH SDS) was − 0.35 ± 1.05 SDS, not significantly different in the three groups (*P* = 0.64). During treatment, rGH titration was performed according to IGF-I SDS and growth response. Mean rGH dose at AH was 0.026 ± 0.009 mg/kg/day with no difference in the three groups (*P* = 0.08).

**Figure 1:**
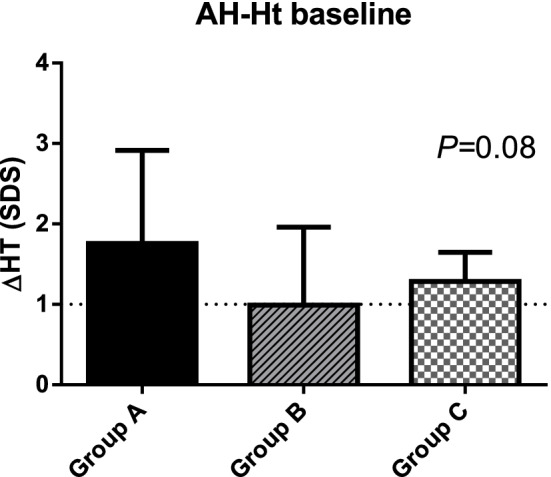
Height gain (∆HT) at adult height (AH) in the three groups of pre-pubertal patients.

In the subgroup of pre-pubertal patients (Group A: n = 17, Group B: n = 13, Group C: n = 10), after a mean time of therapy of 5.7 ± 2.7 years (*P* = 0.17), the mean AH was 163.4 ± 9.5 cm, − 1.22 ± 0.93 SDS (− 0.89 ± 0.99; − 1.54 ± 0.95; − 1.38 ± 0.66 SDS in Group A, B and C, respectively, *P* = 0.14) with an overall ΔHT at AH of 1.37 ± 1.0 SDS (1.95 SDS, 1 SDS and 1.34 in Group A, B and C respectively, *P* = 0.08, showed in Fig. 1). Detailed results are stated in Table 2.

**Table 2:** Response to treatment (pre-pubertal) given as mean (SD) or median (min–max).

	Patients (40)	Group A (17)	Group B (13)	Group C (10)	Sign	n
AH (cm)	163.4 (9.5)	166.2 (9.7)	161.6 (9.8)	160.9 (8.3)	*P* = 0.28	40
AH (SDS)	−1.22 (0.93)	−0.89 (0.99)	−1.54 (0.95)	−1.38 (0.66)	*P* = 0.14	40
MPH (SDS)	−0.81 (0.87)	−0.77 (1.0)	−0.73 (0.79)	−0.99 (0.80)	*P* = 0.77	37
ΔHT at AH (SDS)	1.37 (1.0)	1.95 (−0.39 to 3.79)	1 (− 0.41 to 2.9)	1.34 (0.51–1.7)	*P* = 0.08	36
AH-MPH (SDS)	−0.41 (1.15)	−0.05 (−3.01 to 1.42)	−0.5 (−2.73 to 0.77)	−0.24 (−1.95 to 1.87)	*P* = 0.23	37
Dose AH (mg/kg/day)	0.026 (0.099)	0.03 (0.011)	0.022 (0.008)	0.022 (0.006)	*P* = 0.07	32

*AH* adult height, *MPH* mid-parental height, *HT* height.

**Figure 2:**
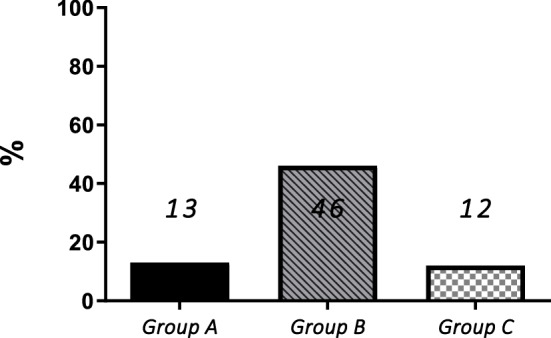
Percentage of “poor responders” according to ∆HT < 1 SDS in the three groups.

**Figure 3:**
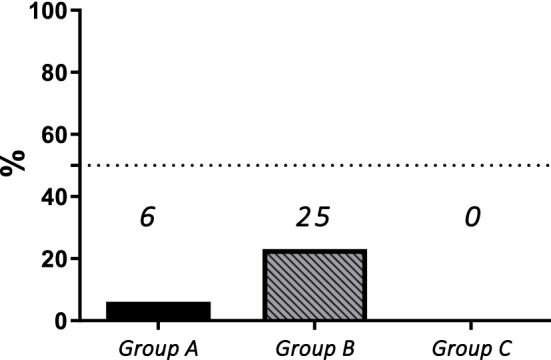
Percentage of patients who failed to achieve mid-parental height (MPH) target in the three groups.

Using the long-term efficacy criteria of Deodati and Cianfarani^35^, Group B showed the highest percentage of “poor responders” with 46% patients with ∆HT AH < 1 SDS (vs 13% and 12% in Group A and C, respectively, showed in Fig. 2) and with 25% children not reaching MPH (vs 6% and 0% in Group A and C, respectively, showed in Fig. 3).

**Figure 4:**
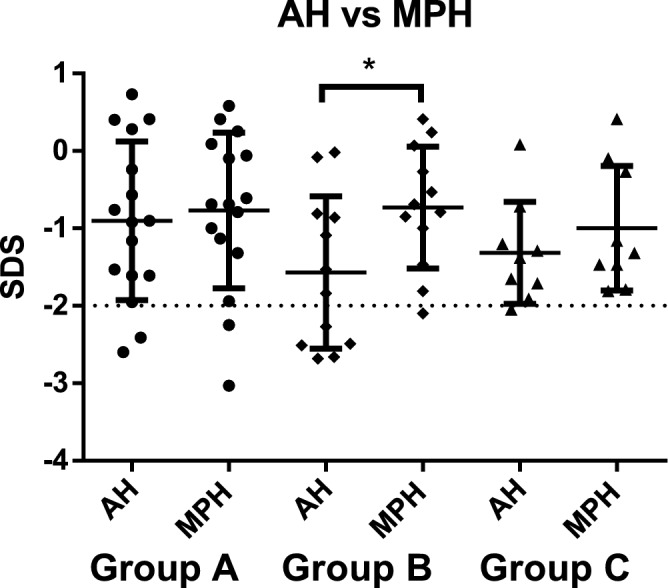
Mean adult height (AH) SDS and mean Mid-parental height (MPH) difference in the three groups. *: *P* = 0.03.

Moreover, comparing mean AH SDS with mean MPH SDS, the difference was statistically significant only for patients of Group B (*P* = 0.03, shown in Fig. 4).

**Figure 5:**
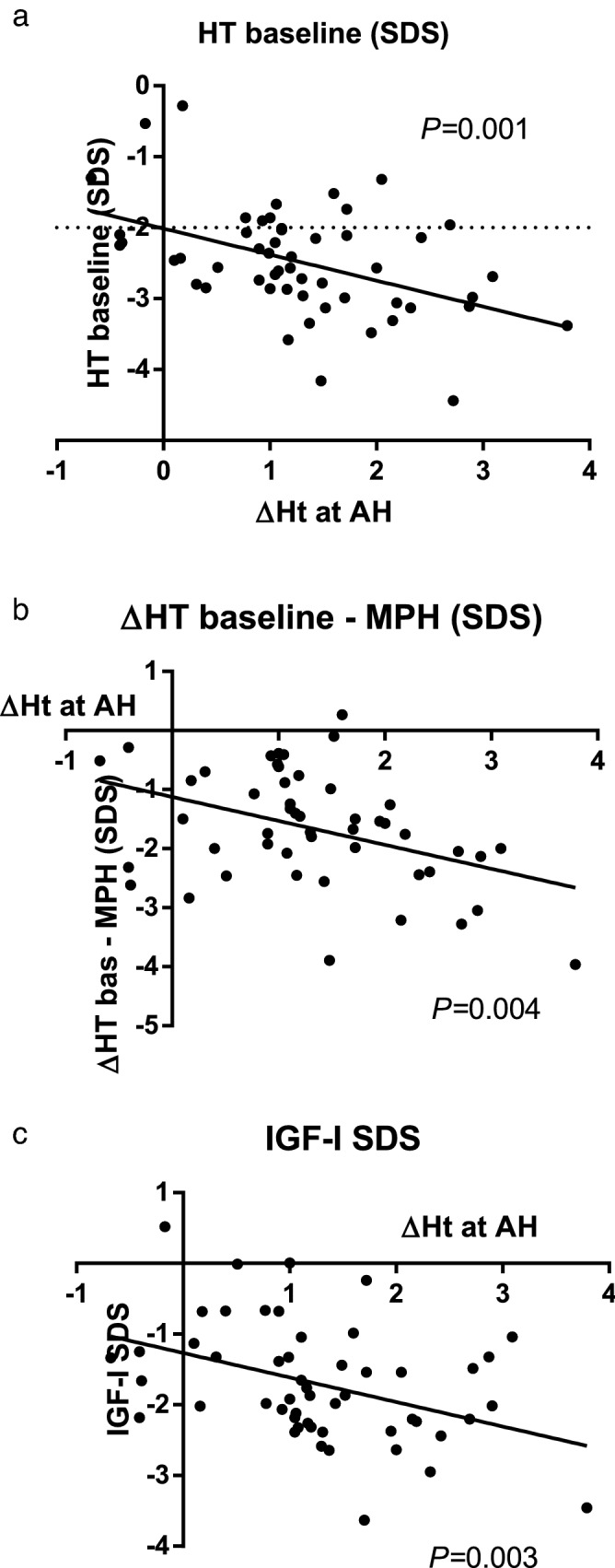
Correlations between height gain (∆HT) at adult height (AH) and (**a**) height SDS at baseline; (**b**) mid-parental height (MPH) difference at baseline expressed as height SDS; (**c**) insulin-like growth factor-I (IGF-I) at baseline.

Height gain at AH was negatively correlated with HT SDS at baseline (*P* = 0.001, showed in Fig. 5a), MPH difference (*P* = 0.004, showed in Fig. 5b), IGF-I SDS at baseline (*P* = 0.003, showed in Fig. 5c) and positively correlated with rGH dose both during the first year of treatment (*P* = 0.032) and at AH (*P* = 0.006, Table 3). At backward stepwise regression model, the most important predictive factors of ∆HT at AH were low HT SDS at baseline (*B* − 0.64 ± 0.22, *P* = 0.007) and rGH dose at AH (*B* 47.4 ± 16.3, *P* = 0.006).

**Table 3:** Regression analysis data on correlations between height gain at adult height and other variables.

ΔHT at AH	*P* value	*r*	*R*^2^
IGF-I (SDS)	0.003	−0.398	0.159
HT baseline (SDS)	0.001	−0.456	0.208
ΔHT baseline-MPH (SDS)	0.004	−0.405	0.164
Dose rGH I year (mg/kg/day)	0.032	0.321	0.103
Dose rGH at AH (mg/kg/day)	0.006	0.414	0.171

*HT* height, *AH* adult height, *IGF-I* insulin-like growth factor-I, *MPH* mid-parental height, *rGH* recombinant growth hormone.

At AH attainment, GHD was reconfirmed in 28% (7/25), 10% (2/19) and 8% (1/13) patients in Group A, B and C, respectively (*P* = 0.18). Among patients with persistent GHD, 8/10 (80%) have minor abnormalities of the pituitary region: 2/10 a partial empty sella and 6/10 an anterior pituitary hypoplasia. Median age at diagnosis of patients with persistent GHD was 12.5 years (range 2.8 to 15.5 years, vs median age in non-persistent GHD of 11.8 years, *P* = 0.71) and mean IGF-I SDS − 1.13 SDS (range − 2.85 to 0.75 SDS, vs median IGF-I SDS − 0.75 in non-persistent GHD, *P* = 0.54).

## Discussion

To the best of our knowledge this is the first paper comparing long-term rGH outcomes (AH, ∆HT at AH and AH-MPH) between GHD children with max peak GH at diagnosis < 8 µg/L and between 8 and 10 µg/L, including also patients with abnormal overnight GH profile. The main results of the present study are that children with lower GH peak at diagnosis have a better response to rGH treatment and more probability of persistent GHD.

To date, there are no new data regarding the normal range for stimulated GH levels and no randomized controlled studies that correlate GH provocative testing results with subsequent long-term efficacy of rGH treatment. Available evidence is only derived from short-term response (in the first few years) and consistently shows some predictive value of GH peaks < 10 μg/L^36–38^. A recent retrospective study on short- and long-term response to treatment in GHD Italian patients reported an higher gain in height at AH in patients with severe GHD defined for GH peak < 5 µg/L (1.85 ± 0.6 SDS) than in patients with peak GH between 5 and 10 µg/L (1.39 ± 0.6 SDS)^39^.

As abovementioned, with the advent of monoclonal antibody GH measurement and newer standards, the cut-offs for GH deficiency should be probably reduced to minimize false-positive results that can mistakenly classify as deficient a child with ISS. This change has already occurred in some European countries where the threshold has been reduced to 7 μg/L. Adjustment of this cut-off should be defined locally by the paediatric endocrinology society of each country^3^. In Italy, the revision of Note 39 in 2014, reducing the threshold at provocative tests from 10 to 8 µg/L, can be considered a first attempt in that sense. However, so far no study has assessed the long-term outcomes differences between patients with GH peak < 8 µg/L or between 8 and 10 µg/L, in order to support the reduction of the diagnostic cut-off.

According to our results, the reduction of GH peak cut-off may help to identify children that will benefit from rGH therapy, discriminating between “good” and “poor responders” not only in terms of median ΔHT at AH (i.e. percentage of patients “poor responders” according to Deodati & Cianfarani criteria), but also in the percentage of patients reaching MPH, an aspect to take into consideration in an expensive subcutaneous daily injective therapy as rGH.

In Group B, indeed, 46% patients showed a dissatisfactory growth response at AH, with 25% “true non-responders” not reaching MPH target at AH. According to the revised biochemical criteria for GHD diagnosis, those patients are now classified as ISS.

Noteworthy, from 2003 on, rGH treatment has been approved from the Food and Drug Administration (FDA) in the United States for children with ISS with HT < − 2.25 SD. In Europe, instead, the European Medicines Agency (EMA) has not extensively approved rGH therapy in ISS and treatment can be only addressed locally to selected patients and under specific circumstances. Although rGH therapy in ISS is yet a matter of debate, the dosages currently used are higher than the ones of GHD patients^40^ and allow, according to three randomized trials, an overall height gain of 1.20 SDS (7.2 cm) in treated children versus 0.34 SDS (2.0 cm) in untreated ones^35^. Thus, the dosages currently used in ISS can lead to question whether the results at AH in our patients belonging to Group B would have been superior with higher rGH doses, as in Group C. Indeed, lower dose rGH treatment in children who would otherwise be making their own pubertal growth spurt actually reduces pubertal growth attainment.

Moreover, based on our results, the reduction of the cut-off seems to be correlated with GHD persistence through transition, as previously found in another report, though using a different threshold (GH < 5 ng/mL). In this work, authors concluded that GH response at provocative tests could be a reliable predictor of persistent GHD^41^. Indeed, in literature it is reported that the 60–80% of patients with childhood-onset GHD re-test normal at provocative tests performed after discontinuation of treatment at AH attainment^23–27^. There are several potential causes of GH response normalization ranging from transient GHD, physiological improvement of hypothalamic-pituitary functions after puberty, NSD with a normal response to the provocative test but altered spontaneous release of GH or poor reproducibility of the GH provocative test^42^. According to our data, the percentage of persistent GHD in Group A (28%) is consistent with the reported prevalence in literature. The lower percentage found in Group B (only 10%), instead, leads to wonder whether all the patients in this Group were true GHD or instead ISS.

On the other hand, GH impairment due to hypothalamic derangement (NSD) is still a controversial issue. Given the complexity of the GH-IGF-I axis, a disruption at any level could result in abnormalities in GH secretion causing poor linear growth and short stature. Non-classical GHD due to NSD was firstly observed in cranio-irradiated children, broadening our understanding of GH deficiencies^33^. Even if the diagnosis of NSD in the absence of a history of cranial irradiation is uncommon, there are some patients with a clinical presentation strongly consistent with GHD that show a good response to rGH therapy, despite their stimulated GH peak being higher than the traditional cut-offs^7^.

In the present cohort, Group C showed a satisfactory response at AH, better than Group B, with all patients reaching MPH target at the end of treatment, even though initially treated with higher rGH dosages. Despite the fact that in patients diagnosed as NSD short-term acceleration of growth was observed in some reports, similar to that seen in children with conventionally defined GHD^33, 43, 44^, neither long-term growth nor AH data were presented. It should be pointed out that the first-year acceleration of height velocity can represent an unreliable predictor of HT gain at AH, especially when chronological age is consistent with pubertal onset and bone age is delayed. The only data available about NSD long-term response to rGH are found in one retrospective study by Radetti and colleagues that showed a mean HT gain of 1.03 SDS at AH, lower than patients with either subnormal levels at GH provocative tests and GH profile (1.85 SDS) but higher than patients with insufficient levels after pharmacological stimulation and normal GH profile (0.49 SDS)^30^.

Moreover, NSD represents one of the possible causes of GH secretion normalization at AH attainment. According to our results, as expected, the majority of patients showed a normal response to the GHRH + arginine test except one (1/13, 8%). In the presence of an underlying hypothalamic derangement, the GHRH + ariginine test is the one with the highest false negative rate, as observed in childhood cancer survivor patients^45–47^. It would be interesting to verify whether using another provocative test (i.e. ITT) the results would have been different.

Assuming the open issues about biochemical diagnosis of GHD, multiple regression analysis of our data confirmed the importance of the auxological criteria reported in literature^48^, as the better response was found in patients with severe short stature at baseline. The regression analysis high-lightened also the importance of rGH dosage, leading to question whether less severe GHD, as well as ISS, has to be treated with higher doses (at least 0.035 mg/kg/day) to show a long-term good response^35, 49^.

Moreover, 80% patients with persistent GHD at transition had anterior pituitary hypoplasia or partial empty sella, suggesting a possible predictive value of MRI findings. In particular, all the patients with persistent GHD of Group B and C had pituitary hypoplasia in accordance with previous data^23^, though literature has reported contrasting results on the topic^41^.

The present retrospective study has some limits: first of all, the relatively high median chronological age at diagnosis (11.9 years). In order to avoid biases related to delayed diagnosis, we analysed long-term outcomes in the subgroup of patients who were pre-pubertal at baseline. Even after that, it is possible an overlapping of GHD in constitutional delay of growth and puberty in our population, given the difficulties sometimes encountered in the differential diagnosis and the lack of an agreement on the use of sex hormone priming^3^. Secondly, given the wide span time of the study, it has been impossible to assess GH concentrations with the same assay, though in our central laboratory only one change occurred in 2007, as stated in the “methods” section.

Moreover, retesting has been performed with the use of GHRH + arginine in the vast majority of patients (n = 54): this combined test represents one of the most powerful GH stimulation tests showing less intra-individual variability, but remains questionable in several patients, especially in the presence of primary hypothalamic dysfunction, as in childhood cancer survivors or NSD, as abovementioned^50^.

In conclusion, present data demonstrate that a reduction of diagnostic cut-off at GH stimulation tests, as suggested by the latest recommendations^3^, could better discriminate between “good” and “poor responders” and predict the persistence of GHD through transition. On the other hand, GHD diagnosis solely based on provocative tests could exclude patients that might benefit from rGH treatment. Low baseline IGF-I SDS and long-term outcomes in patients with normal stimulation test bring back to light NSD as a possible aetiology of ISS presenting a good response to therapy.
